# Social network and HIV/AIDS: A bibliometric analysis of global literature

**DOI:** 10.3389/fpubh.2022.1015023

**Published:** 2022-11-02

**Authors:** Linh Phuong Doan, Long Hoang Nguyen, Pascal Auquier, Laurent Boyer, Guillaume Fond, Hien Thu Nguyen, Carl A. Latkin, Giang Thu Vu, Brian J. Hall, Cyrus S. H. Ho, Roger C. M. Ho

**Affiliations:** ^1^Institute for Global Health Innovations, Duy Tan University, Da Nang, Vietnam; ^2^Faculty of Medicine, Duy Tan University, Da Nang, Vietnam; ^3^Department of Global Public Health, Karolinska Institute, Stockholm, Sweden; ^4^Research Center on Health Services and Quality of Life, Aix Marseille University, Marseille, France; ^5^Bloomberg School of Public Health, Johns Hopkins University, Baltimore, MD, United States; ^6^Center of Excellence in Health Services and System Research, Nguyen Tat Thanh University, Ho Chi Minh, Vietnam; ^7^School of Global Public Health, New York University, New York, NY, United States; ^8^Department of Psychological Medicine, Yong Loo Lin School of Medicine, National University of Singapore, Singapore, Singapore; ^9^Institute for Health Innovation and Technology (iHealthtech), National University of Singapore, Singapore, Singapore

**Keywords:** social network, HIV, bibliometric, topic modeling, Latent Dirichlet Allocation

## Abstract

Social networks (SN) shape HIV risk behaviors and transmission. This study was performed to quantify research development, patterns, and trends in the use of SN in the field of HIV/AIDS, and used Global publications extracted from the Web of Science Core Collection database. Networks of countries, research disciplines, and most frequently used terms were visualized. The Latent Dirichlet Allocation method was used for topic modeling. A linear regression model was utilized to identify the trend of research development. During the period 1991–2019, in a total of 5,698 publications, topics with the highest volume of publications consisted of (1) mental disorders (16.1%); (2) HIV/sexually transmitted infections prevalence in key populations (9.9%); and (3) HIV-related stigma (9.3%). Discrepancies in the geographical distribution of publications were also observed. This study highlighted (1) the rapid growth of publications on a wide range of topics regarding SN in the field of HIV/AIDS, and (2) the importance of SN in HIV prevention, treatment, and care. The findings of this study suggest the need for interventions using SN and the improvement of research capacity *via* regional collaborations to reduce the HIV burden in low- and middle-income countries.

## Introduction

Recognition of social networks' role in shaping HIV risk behaviors and transmission is substantially increasing in global research (1–4). The first evidence of this role of social networks (SN) appeared in 1984, which was about the transmission of AIDS *via* partner links among 40 men who have sex with men (MSM) living with AIDS (5). Recent data indicated that individual-level behaviors are unable to comprehensively explain the risks of acquiring HIV in higher-risk populations (6). Considering the social environment or SN when developing interventions is thus necessary to have a greater influence on behavioral change rather than only focusing on individual factors (7).

SN is defined as a set of linkages among individuals sharing similar interests or having interpersonal interactions (8). Impacts of SN on health behaviors and outcomes depend on its structural (e.g., size, density, degree, betweenness, centrality, and homogeneity) (7, 9–11), and functional (e.g., social support or social capital) characteristics (12–14). Understanding an individual's network characteristics is useful to inform the dynamic spread of disease (1–4, 15, 16), and provides important implications for efficiently developing interventions as well as allocating resources for HIV prevention (17). Previous studies suggest that people with large network sizes have a greater possibility of HIV exposure from risk practices from network members than people with smaller network sizes (18, 19). For example, among key populations such as people who inject drugs (PWIDs) and MSM, larger risk networks contribute to needle sharing, unprotected sexual practice, and poor outcomes in HIV treatment (11, 20, 21). In addition, risk or protective behaviors frequently occur in a dense network where these behaviors are normalized or encouraged (18). Studies in PWIDs or migrants showed that condom use practice significantly increased with a positive attitude toward condom use among network members (22, 23). In terms of functional aspects, previous research indicated that social support was associated with engagement in harm reduction interventions (24–26), as well as health improvement in HIV care (27).

In HIV/AIDS research, SN interventions (SNIs) have been successful in reaching and improving HIV-related risk behaviors among hidden or hard-to-reach populations [e.g., PWIDs, MSM, commercial sex workers (CSWs), or other sexual minority groups] (28–31). Moreover, a study in Thailand and the United States indicated that behavioral change could be sustained for more than 2 years (32). These results imply the need to incorporate the SN component in HIV interventions among key groups for both roles: to understand the sources of virus transmission and to serve as a channel to disseminate information for HIV prevention and treatment (33, 34).

The importance of SN has attracted scholars around the world to investigate its application in mitigating the burden of HIV/AIDS. There has been substantial growth in the body of publications about this topic over decades, and many systematic reviews have been performed to measure the effects of SN and SNIs on different key populations (3, 27, 31, 35–38). However, these reviews have not been able to reflect the entirety of publications to show research trends, patterns, and landscapes about SN in the field of HIV. In order to fill existing knowledge gaps, this bibliometric study was performed to analyze the research development, patterns, and trends in the use of SN in the field of HIV/AIDS.

## Materials and methods

### Searching strategy and eligibility criteria

In our study, the Web of Science (WoS) Core Collection database was chosen for the searching process because it includes more information for our analysis than other databases including publications' contents (e.g., title, abstract, keyword, and research discipline) and metrics (e.g., citations and download times) (39, 40). The Web of Science database was chosen for our analysis because it is valuable for analyzing research fields, something that Scopus, PubMed, and other databases are unable to do. The WOS database also included superior scientific journals, whereas other databases only included articles of varying quality. In order to evaluate the research productivity in various subgroups, we were also able to conduct sophisticated searches and filter the results based on predetermined criteria thanks to the WOS. Additionally, the WOS supports a number of analytic metrics and has a high citation report coverage, both of which help with bibliometric analysis of already published works.

We developed the search query aiming to retrieve the articles about social networks application in HIV research. We divided the search process into two stages. First, we used the search terms “HIV,” “AIDS,” “Human Immunodeficiency Virus,” and “Acquired Immune Deficiency Syndrome” for the topic search. Then, after achieving the HIV/AIDS dataset, we searched the following terms in the title/abstract to filter the social network-related studies: “social network(s),” “social network analysis,” “network analysis,” “network analyses,” “friendship network(s),” “peer network(s),” “sociometric(s),” “sociogram(s),” “sociomap(s),” “egonetwork(s),” “respondent driven,” “respondent-driven,” “social support,” “interpersonal,” “cliques,” “community support,” and “social capital.” We also searched for terms regarding popular software for social network analysis such as “UCINET,” “NetDraw,” and “Pajek.”

**Figure 1:**
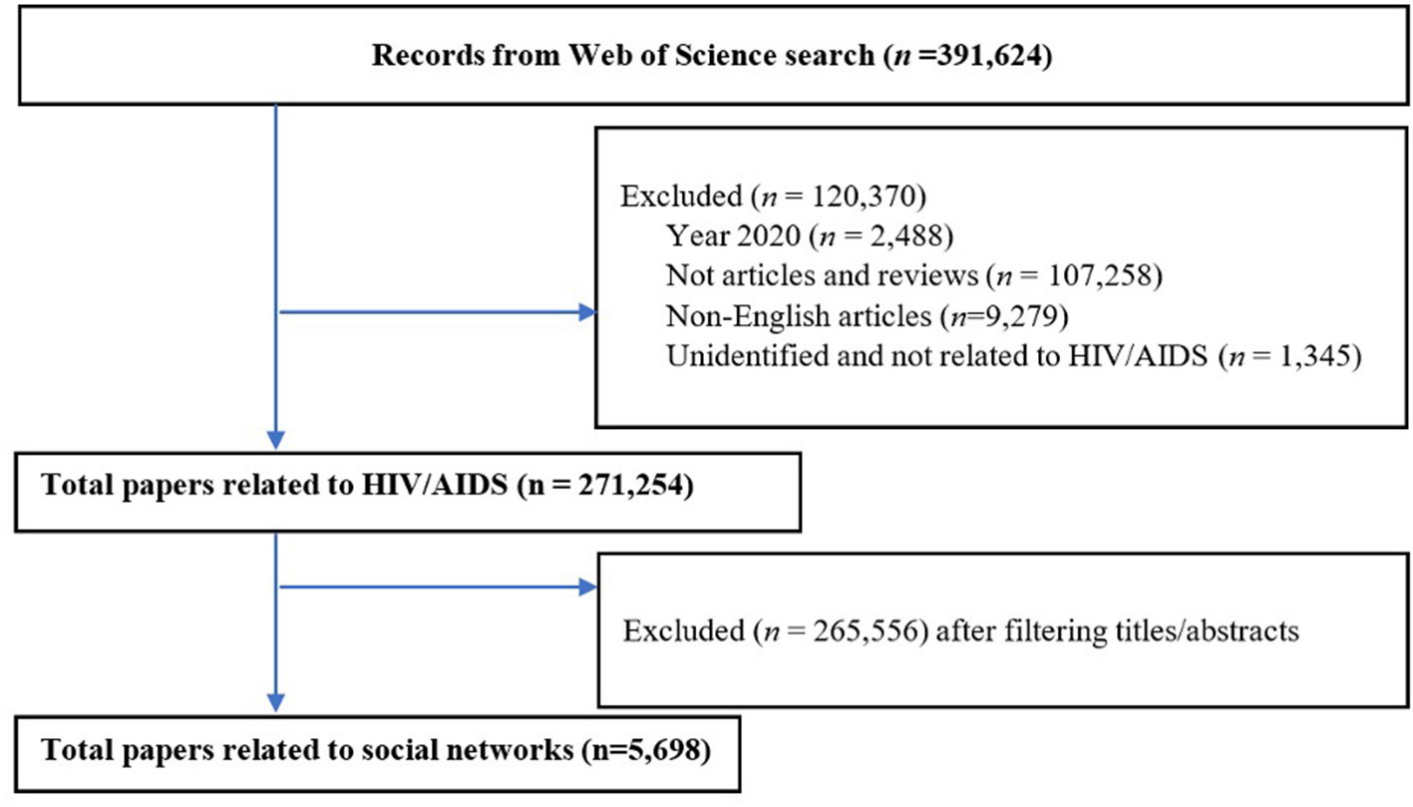
Flow chart of the search process.

Only articles were (1) original or review articles, (2) published in peer-reviewed journals in the English language, and (3) used or discussed the social networks in HIV-related topics were included. We excluded gray literature, book/book chapter, conference abstract/proceeding, or other types of documents that did not meet the inclusion criteria. Finally, a total of 5,698 articles were used for bibliometric analysis (Figure 1).

### Study design and statistical analysis

We applied the bibliometric approach to evaluate the selected articles. For bibliometric analysis, we used Stata version 15.0^1^ (Stata Corp., Texas, United States) to perform basic descriptive statistics for publication year, the number of papers/per year, total citations up to 2019, mean citation rate per year, total usage in the last 6 months/5 years, and mean use rate in the last 6 months/5 years.

For visualizing the co-occurrence of the most frequent terms, the VOSviewer^2^ (version 1.6.8, Center for Science and Technology, Leiden University, the Netherlands) software was used to illustrate the networks of 226 terms, which appeared at least 100 times. In addition, this software was used to present the research disciplines and countries of collaboration among the selected publications.

We used Latent Dirichlet Allocation (LDA), a type of machine learning method, to exploit the latent topics and figure out the research tendency over the years. LDA is one of the most used techniques in this area for further analysis. In order to understand the structure of research development, current trends, and multidisciplinary landscapes of research in HIV/AIDS and social networks, it was useful to group articles into subjects that were comparable to one another. Each component in a random vector represents the likelihood of drawing the words or texts connected with that component. We utilized LDA to classify the text in each abstract to a topic where Dirichlet is employed as a distribution over discrete distribution (41–43). The scientific fields were divided into corresponding groups using principal component analysis (PCA). As a result, we could use LDA to annotate the topic of the papers to find hidden themes and gain a comprehensive understanding of the patterns of HIV/AIDS and social networks (44). A total of 15 topics emerged based on consultation with HIV and social network experts (45). Each topic was labeled by referring to the topic's 15 most frequent terms as well as titles/abstracts within the topic. We then computed the volume of publications per topic per year, as well as the share of publications across topics within each year. We performed the linear regression model, with the share of publication as the dependent variable and the number of years as the independent variable, to examine the research tendency in general and in different time intervals (1991–2000, 2001–2010, and 2011–2019). A topic was recognized as a “hot” topic if the coefficient had a significantly positive linear trend, while a topic was “cold” if the coefficient had a significantly negative linear trend. We used a *p*-value < 0.05 to detect statistical significance.

## Results

The general characteristics of selected articles are presented in Table 1. During the period 1991–2019, there were a total of 5,698 papers published in English peer-reviewed journals indexed in the WoS database. Overall, the number of publications increased rapidly from 7 articles in 1991 to 560 articles in 2019. The highest number of articles was also recorded in 2019. However, publications in 2012 had the highest number of citations (8,978 citations) and mean use rate in the last 5 years (3.19 downloading times/paper/year). Articles published in 2001 had the highest mean cited rate per year (3.84 cites/paper/year), while those published in 2015 had the highest total usage in the last 5 years (5,908 downloading times). There were 134 active countries, of which 90 countries had five publications or more. The top five clusters with the highest number of countries were the red, green, blue, yellow, and purple clusters, which were led by the United States (3,912 publications), Brazil (110 publications), England (448 publications), Iran (34 publications) and China (365 publications), respectively (see Image, Supplemental Digital Content 1, which indicates the geographical collaborations among countries). Publications on the social network use in HIV research involved 112 disciplines, of which concentrated on several major disciplines including “Public, Environmental & Occupational Health,” “Health Policy & Services,” “Pharmacology & Pharmacy,” “Obstetrics & Gynecology,” “Immunology,” and “Engineering, Electrical & Electronic” (see Image, Supplemental Digital Content 2, which visualizes the co-occurrence of research disciplines in selected publications. The circle's size is based on the link strength, while the line reflects the co-occurrence of disciplines).

**Table 1:** General characteristics of publications.

**Year published**	**Total number of papers**	**Total citations**	**Mean cite rate per year**	**Total usage last 6 month**	**Total usage last 5 years**	**Mean use rate last 6 month**	**Mean use rate last 5 year**
2019	560	494	0.88	918	1,956	1.64	0.70
2018	547	1,781	1.63	603	3,696	1.10	1.35
2017	501	3,383	2.25	281	3,742	0.56	1.49
2016	486	4,445	2.29	242	5,073	0.50	2.09
2015	499	6,825	2.74	216	5,908	0.43	2.37
2014	399	8,020	3.35	169	5,887	0.42	2.95
2013	365	7,849	3.07	131	5,642	0.36	3.09
2012	316	8,978	3.55	123	5,041	0.39	3.19
2011	286	8,297	3.22	79	2,962	0.28	2.07
2010	206	6,476	3.14	47	1,956	0.23	1.90
2009	222	8,284	3.39	74	2,201	0.33	1.98
2008	172	7,490	3.63	51	1,734	0.30	2.02
2007	141	6,686	3.65	35	1,264	0.25	1.79
2006	126	5,573	3.16	36	936	0.29	1.49
2005	99	5,348	3.60	30	963	0.30	1.95
2004	69	3,727	3.38	22	483	0.32	1.40
2003	87	4,655	3.15	26	740	0.30	1.70
2002	81	4,642	3.18	19	621	0.23	1.53
2001	72	5,253	3.84	29	637	0.40	1.77
2000	65	3,996	3.07	17	522	0.26	1.61
1999	53	3,310	2.97	12	468	0.23	1.77
1998	62	2,339	1.71	14	349	0.23	1.13
1997	57	2,868	2.19	11	341	0.19	1.20
1996	65	2,959	1.90	12	288	0.18	0.89
1995	42	1,420	1.35	5	146	0.12	0.70
1994	51	2,776	2.09	13	247	0.25	0.97
1993	39	2,742	2.60	9	227	0.23	1.16
1992	23	1,399	2.17	8	86	0.35	0.75
1991	7	295	1.45	1	22	0.14	0.63

Results of twenty papers with the highest number of citations (see Table, Supplemental Digital Content 2, which showed the twenty most-cited papers) showed that the majority of articles mentioned cross-sectional studies, and only one intervention was included on the list. The article with the highest volume of citations was “Explaining the limited effectiveness of legalistic remedies for trust distrust” (46), which aimed to build interpersonal trust in organizations and illustrate the theory by using the case study about organizational responses to HIV-positive employees. The second paper entitled “Measuring stigma in people with HIV: Psychometric assessment of the HIV stigma scale” aimed to evaluate the psychometric properties of a stigma-related instrument (47). The domain scores were found to be associated with the degree of social support and social conflict (47). The third paper was a scoping review to provide synthesized evidence about the sampling method for reaching hidden populations in HIV surveys. This paper mentioned the respondent-driven sampling method, which is a social network-based sampling approach (48).

**Figure 2:**
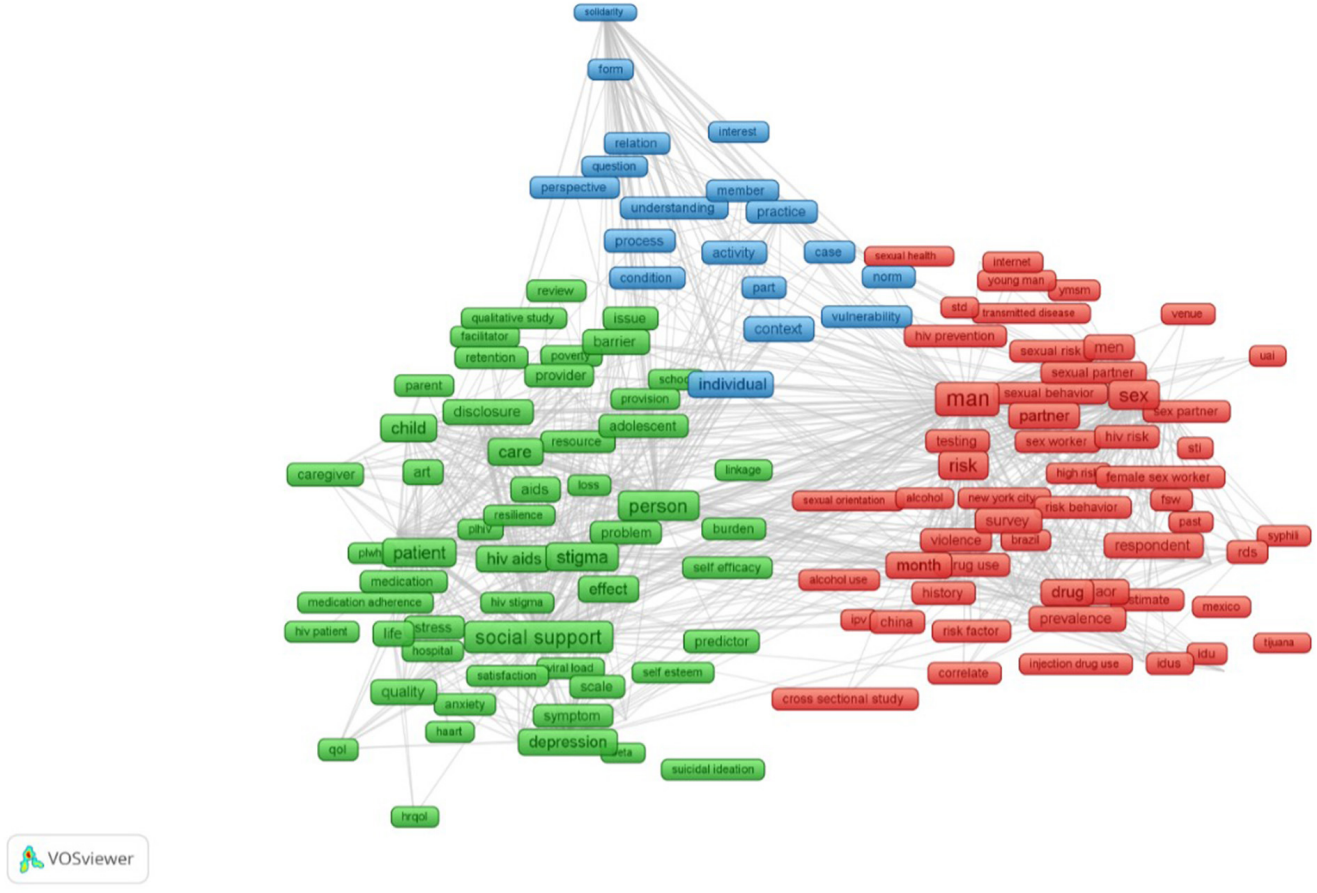
Co-occurrence of most frequent terms in titles and abstracts.

The results of the content analysis are illustrated in Figure 2. Three major clusters of terms were found. The green cluster showed the studies exploring the role of SN and social support in reducing stigma and discrimination, improving mental disorders (e.g., depression, anxiety, stress, suicidal ideation), and HIV care (e.g., medication adherence, patient, hospital) in patients, infants, and caregivers. The red cluster revealed studies measuring the effects of SN on risk behaviors (e.g., sexual risk, injection drug use, alcohol), HIV and sexually transmitted infections (STIs) testing (e.g., testing, transmitted disease), and violence (e.g., violence, intimate partner violence – IPV) among youths, MSM, drug users, and sex workers. Finally, the blue cluster depicts studies mentioning the social capital, social cohesion, and social norms about HIV-related behaviors among vulnerable populations to HIV.

Fifteen topics as outputs of the LDA model are presented in Table 2. Each topic was labeled by referring to the topic's 15 most frequent terms as well as titles/abstracts within a topic. Topics with the highest volume of publications consisted of Topic 3 “Impacts of social network and social support on mental disorders” (16.1%), and Topic 14: “Social network and HIV/sexually transmitted infections prevalence in key populations (9.9%)”; and Topic 9 “Social network and social support in coping HIV-related stigma” (9.3%). The percentage of publications in topics 9 and 14 significantly increased, while the share of publications in Topics 3, 6, 8, 10, and 12 had declined over the years. [See Image, Supplemental Digital Content 2, which presents the changes in publication share (%) across topics].

**Table 2:** Fifteen topics are classified by LDA and the 15 most frequent terms in each topic.

**No**.	**Topic**	**Most frequent terms**	***n***	**%**
1	Social network interventions in online social media	intervention; group; participants; prevention; social; online; study; trial; interventions; information; networking; media; control; groups; health	348	6.1%
2	Social networks, illicit drug injection, and viral hepatitis transmission	injection; users; sampling; drugs; injecting; population; inject; study; people; respondent-driven; recruitment; methods; hepatitis; using; sample	308	5.4%
3	Impacts of social networks and social support on mental disorders	support; social; depression; health; stigma; study; symptoms; associated; coping; between; mental; living; psychological; perceived; depressive	915	16.1%
4	Evidence synthesis in social network-related interventions	health; studies; research; interventions; review; social; outcomes; literature; evidence; factors; systematic; countries; prevention; included; identified	256	4.5%
5	Social network improves HIV testing services access and linkage to care	health; services; community; africa; access; south; testing; service; support; treatment; workers; prevention; knowledge; needs; rural	306	5.4%
6	Social network affects HIV care and treatment outcomes	patients; women; infection; immunodeficiency; hiv-infected; virus; human; disease; diagnosis; during; study; results; hospital; medical; diagnosed	184	3.2%
7	Social networks and women's reproductive health	contraceptive; pregnancy; contraception; rural; hombres; republic; reproductive; pregnancies; abortion; hommes; unintended; dominican; relations; plasma; planning	22	0.4%
8	Social network affects sexual risk behaviors and violence	sexual; partners; condom; women; partner; violence; behavior; interpersonal; behaviors; relationship; between; prevention; reported; sexually; unprotected	397	7.0%
9	Social network and social support in coping with HIV-related stigma	stigma; health; social; women; support; qualitative; interviews; living; study; experiences; participants; barriers; conducted; discrimination; people	530	9.3%
10	Social and family support to vulnerable populations with HIV-positive	children; support; disclosure; family; adolescents; social; status; caregivers; youth; hiv/aids; families; living; study; child; parents	216	3.8%
11	Social network and HIV risk behaviors in homeless and immigrant people	social; network; networks; behaviors; associated; african; alcohol; substance; black; american; study; characteristics; between; members; homeless	364	6.4%
12	Social capital in HIV prevention and care	social; capital; community; hiv/aids; networks; communities; context; solidarity; people; paper; structural; groups; group; within; health	254	4.5%
13	Social network modeling of HIV/AIDS transmission	network; analysis; transmission; networks; hiv-1; individuals; infection; between; using; human; structure; resistance; virus; clusters; disease	198	3.5%
14	Social network and HIV/sexually transmitted infections prevalence in key populations	prevalence; testing; associated; infection; factors; reported; using; sampling; study; participants; years; having; female; workers; results	566	9.9%
15	Impacts of social network and social support on HIV treatment adherence	adherence; treatment; antiretroviral; patients; therapy; support; medication; viral; factors; study; outcomes; associated; retention; patient; hiv-infected	264	4.6%

The results of the linear regression model are depicted in Table 3. Based on the coefficient, we classified topics into two categories, namely “hot” and “cold.” In the period 1991–2000, topic 15 “Impacts of social network and social support on HIV treatment adherence” was found to be a “hot” topic since the publication share averaged an increase of 0.52% per year. Meanwhile, in the period 2001–2010, the research focus shifted to topic 14 “Social network and HIV/sexually transmitted infections prevalence in key populations” with an increase of 1.42% in the publication share per year. However, the share of publications in Topic 1 “Social network interventions in online social media” and Topic 3 “Impacts of social network and social support on mental disorders” reduced significantly. From 2011–2019, topic 3 was considered a “hot” topic along with topic 9 “Social network and social support in coping HIV-related stigma,” while topics 8, 11, and 12 witnessed a downward trend in the publication share. In the whole period 1991–2019, there was an upward trend in the publication share of topic 2 “Social network, illicit drug injection and viral hepatitis transmission,” topic 4 “Evidence synthesis in social network-related interventions,” topic 7 “Social network and women's reproductive health,” topic 9, topic 14 and topic 15, while topic 3, 6 and 8 had a significantly declining tendency.

**Table 3:** Hot/Cold topics.

**Topic**	**1991–2000**		**2001–2010**		**2011–2019**		**Overall**	
	**Coefficient**	**Hot/Cold**	**Coefficient**	**Hot/Cold**	**Coefficient**	**Hot/Cold**	**Coefficient**	**Hot/Cold**
1	0.78	–	−0.58^*^	Cold	0.09	–	0.11	–
2	0.30	–	0.49	–	−0.20	–	0.25^*^	Hot
3	−0.31	–	−1.57^*^	Cold	0.73^*^	Hot	−0.66^*^	Cold
4	0.15	–	0.19	–	0.28	–	0.09^*^	Hot
5	−1.36	–	0.29	–	0.01	–	−0.09	–
6	−0.96	–	−0.03	–	0.10	–	−0.42^*^	Cold
7	–	–	−0.04	–	0.10	–	0.02^*^	Hot
8	−1.23	–	−0.38	–	−0.78^*^	Cold	−0.45^*^	Cold
9	−0.15	–	0.27	–	1.18^*^	Hot	0.32^*^	Hot
10	0.43	–	0.08	–	−0.37	–	−0.02	–
11	0.77	–	0.13	–	−0.44^*^	Cold	0.11	–
12	0.66	–	0.03	–	−0.57^*^	Cold	−0.11	–
13	0.38	–	−0.23	–	−0.02	–	0.04	–
14	0.01	–	1.42^*^	Hot	0.14	–	0.61^*^	Hot
15	0.52^*^	Hot	−0.18	–	−0.26	–	0.20^*^	Hot

* P-value < 0.05.

## Discussion

This bibliometric study characterized the global status of SN application in HIV research. The result indicated a substantial increase in publications and the involvement of multiple disciplines to address the SN-related research questions. Our findings also offered an insight into the SN usage in the field of HIV for approximately 30 years by uncovering the latent topics and identifying the shift of research focus at different time intervals. This approach allowed us to detect knowledge gaps and develop the research agenda for enhancing the use of SN in HIV topics.

The SN usage in HIV/AIDS gained great attention from scholars around the world, which was shown *via* the exponential growth in the number of publications, citations, and usage during the period 1991–2019. This finding is particularly important at a time when the global funding for HIV/AIDS is reduced in recent years, which might diminish resources for HIV prevention interventions across countries (49). Previous reviews indicate that SN is an essential component in both sampling and intervention perspectives for alleviating HIV risk behaviors and transmission (3, 27, 31, 35–38), and improving mental health (50) and quality of life (51, 52). SN-based sampling approaches (e.g., respondent-driven sampling - RDS) are low-cost and feasible methods to recruit at-risk populations on a large scale (48). Moreover, SNIs can be more cost-effective and sustained than individual-focused interventions given they take into account the influence of social and environmental factors in behavioral changes (28–31, 35, 53). The findings of this study supplemented the prior reviews to confirm the promising role of SN-based approaches, which can be helpful for researchers, policy-makers, and foreign donors in the world to develop the priorities in HIV research in the future.

Along with the rapid growth of publications, our analysis indicated that SN-related studies had the contribution of researchers from different disciplines, even in non-medical fields such as engineering or mathematics. This phenomenon can be explained by the fact that improving SN-based approaches requires the involvement of these scientists to optimize the SN-related parameters *via* simulation/modeling or fieldwork. Moreover, the advancement of technology offers various tools to enhance the performance of SN-based approaches (such as RDS software for sampling, SN software to visualize the networks of higher-risk populations, online social network sites, or mobile phone applications for interventions) (54–56), which also demands the collaborations among scientists from different areas.

In this study, we also observed that the geographical distribution of articles was not equal. Although the result recorded that authors from ninety countries participated in the SN-related studies, most of the publications were produced by researchers from the United States, England, South Africa, Canada, China, and Australia. Meanwhile, there were a limited number of publications in other countries in African (e.g., Burkina Faso, Cameroon, or Congo) or Asian (Myanmar, Indonesia, or Cambodia) countries with a high burden of HIV. Additionally, among approximately 6,000 selected publications, we only found more than 400 papers with intervention design, and most of them were performed in high-income countries. This finding suggested that the application of SN in developing HIV-related interventions is still limited. Moreover, it should be noted that behavioral change interventions rely on the behaviors and cultures of target populations, in other words, must be contextualized in order to obtain the highest effectiveness (57). The limited collaborations may hinder the knowledge translation into practice, raising the need for support from the most productive countries to others to improve the applicability of the evidence in different contexts. Regional collaboration initiatives, with the productive countries as a central role, may be beneficial to the country members by improving the research capacity and evidence quality.

By using the LDA, we could uncover the fifteen latent topics of selected publications. The findings of this study indicated a diversity of topics regarding SN use, including risk behaviors, HIV/STIs prevalence, mental disorders, HIV care and treatment, stigma, reproductive health service use, and online social media interventions. Mental health was the dominant topic, followed by HIV/STI prevalence and HIV-related stigma. This result could be justified by the characteristics of SN that SN reflects how one person can socially interact with another. Social support serves as a functional characteristic of SN, and people with stronger social support can mitigate the negative influence of mental problems such as depression or anxiety (58). Meanwhile, stigma is a socially constructed condition, which depends on different social contexts and the power of stigmatized individuals' relationships (59, 60). The results also help to capture the shift of research focus regarding SN use at different time intervals. While HIV treatment and care were the main topics during the period 1991–2000. This can be explained that this duration was the time when the antiretroviral treatment was widely tested and implemented; thus, ensuring medication adherence was the priority of the funder to ensure that the treatment was effective. In the next decade, from 2001–2010, the research priorities moved to identify the prevalence of HIV/STIs in hidden populations. By using SN data, the donors and governments could estimate the size of HIV populations in order to allocate the appropriate resource for HIV prevention and treatment. After that, mental health and stigma became the “hot” topic in the last decade. This finding can be justified that studies in the period 1991–2010 have well-documented associations between SN with HIV/STI, risk behaviors, and HIV treatment and care; hence, researchers prioritized optimizing other life and social aspects of target populations, including quality of life, mental health, and stigma. However, we found a lack of research on reproductive health care among women (e.g., prevention of mother-to-child transmission, or antenatal care), as well as examining the influence of online relationships on the risk for HIV/AIDS, especially in the era of Internet and smartphone use expansion (61, 62); therefore, future research should be warranted.

Study strengths included the analysis of global literature as well as the application of advanced techniques namely the LDA for content analysis. Nonetheless, some limitations should be noted. First, we only searched literature through only the WoS, which might cover publications in peer-reviewed journals not indexed in this database. Second, gray and non-English literature was excluded from this study. Third, we only performed content analysis *via* titles/abstracts of publications instead of using full texts.

## Conclusion

This study highlighted the rapid growth of publications in a wide range of topics regarding SN in the field of HIV/AIDS, underlining the importance of SN in HIV prevention, treatment, and care. The findings of this study suggested the need for interventions using SN and the improvement of research capacity *via* regional collaborations to reduce the HIV burden in low- and middle-income countries.

## Data availability statement

The original contributions presented in the study are included in the article/Supplementary files, further inquiries can be directed to the corresponding author.

## Author contributions

Conceptualization: LPD, LB, CAL, and BJH. Data curation: LHN, HTN, GTV, and CSHH. Formal analysis: PA, HTN, and BJH. Investigation: PA, CAL, and CSHH. Methodology: PA, GTV, BJH, and RCMH. Supervision: HTN, CAL, and BJH. Writing—original draft: LPD, LHN, LB, GTV, and RCMH. Writing—review and editing: LPD, LHN, LB, CSHH, and RCMH. All authors have read and agreed to the published version of the manuscript.

## Funding

The article process charge of this paper is supported by NUS Department of Psychological Medicine (R-177-000-100-001/R-177-000-003-001) and NUS iHeathtech Other Operating Expenses (R-722-000-004-731).

## Conflict of interest

The authors declare that the research was conducted in the absence of any commercial or financial relationships that could be construed as a potential conflict of interest.

## Publisher's note

All claims expressed in this article are solely those of the authors and do not necessarily represent those of their affiliated organizations, or those of the publisher, the editors and the reviewers. Any product that may be evaluated in this article, or claim that may be made by its manufacturer, is not guaranteed or endorsed by the publisher.

## Footnotes

^1^The Stata software: https://www.stata.com/.

^2^The VOSviewer software: https://www.vosviewer.com/.
