# Supplementary material for: Social network and HIV/AIDS: A bibliometric analysis of global literature
Source: Front Public Health. 2022 Nov 2;10:1015023. doi: 10.3389/fpubh.2022.1015023 (PMC9666395; doi:10.3389/fpubh.2022.1015023)

**
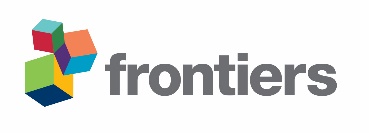
**

**LIST OF SUPPLEMENTAL MATERIAL**

**Supplemental Digital Content 1**


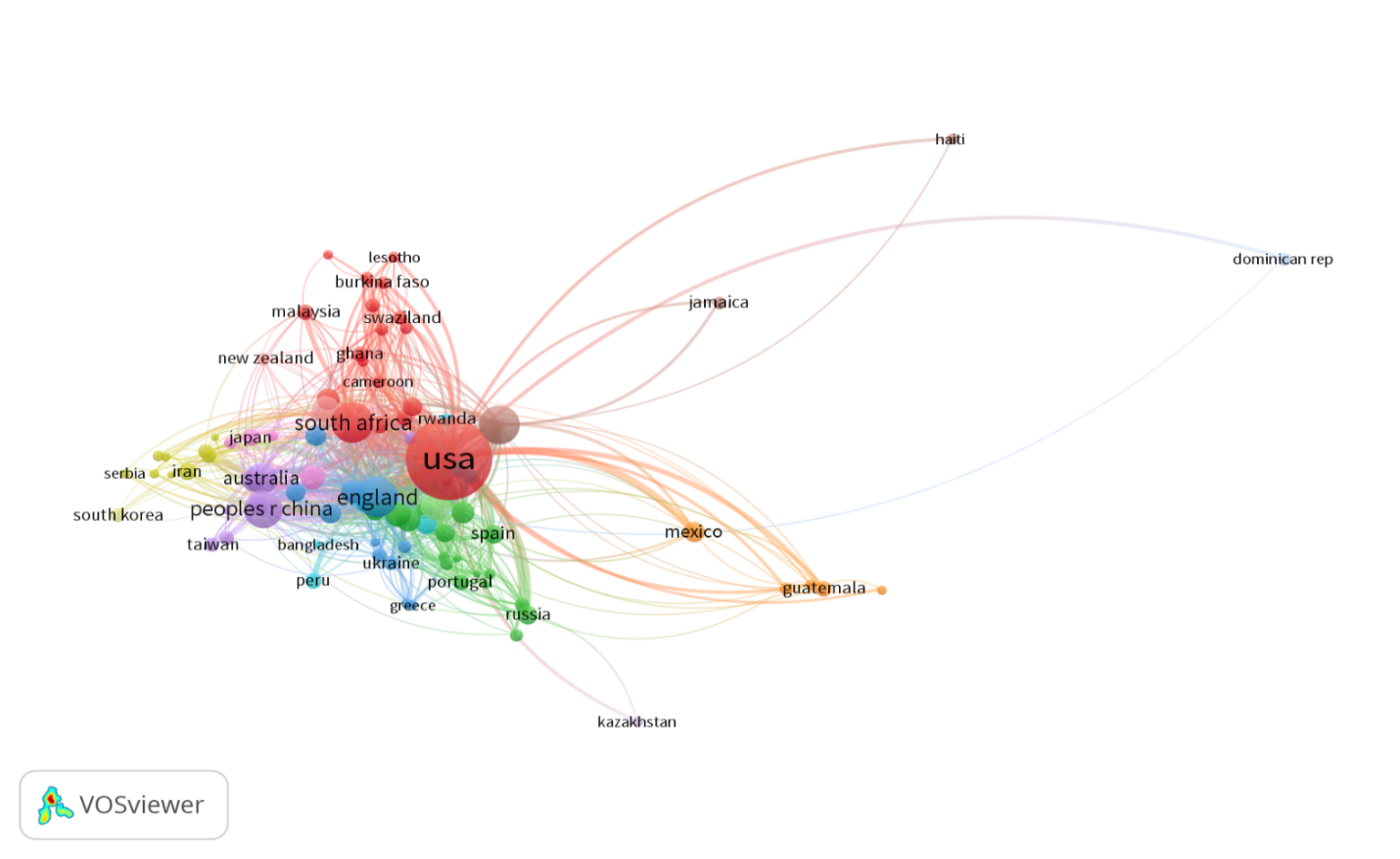


**Supplemental Digital Content 2**


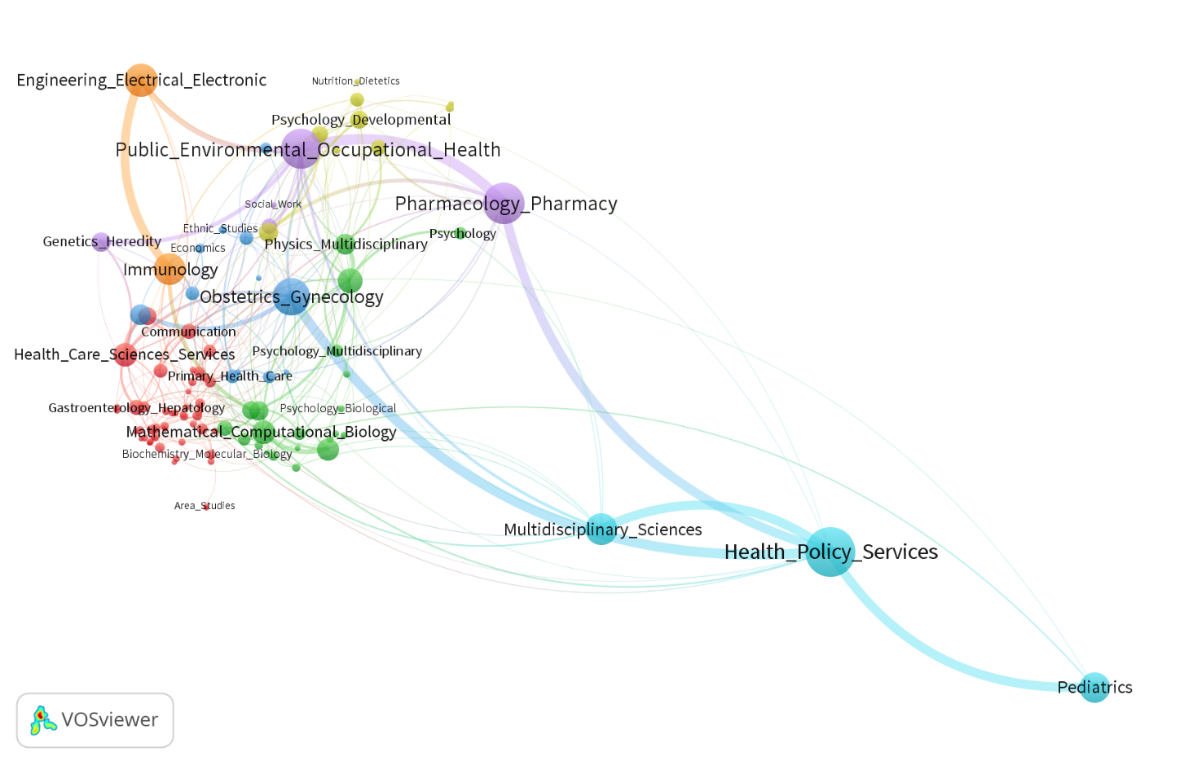


**Supplemental Digital Content 3**

**Table S1. Top 20 most cited papers**

|  | **Title** | **Journal** | **Cite** | **Year** | **Cite rate** |
| --- | --- | --- | --- | --- | --- |
| 1 | Explaining the limited effectiveness of legalistic remedies for trust distrust | Organization Science | 677 | 1993 | 25.1 |
| 2 | Measuring stigma in people with HIV: Psychometric assessment of the HIV stigma scale | Research in Nursing & Health | 657 | 2001 | 34.6 |
| 3 | Review of sampling hard-to-reach and hidden populations for HIV surveillance | AIDS | 628 | 2005 | 41.9 |
| 4 | The social structural production of HIV risk among injecting drug users | Social Science & Medicine | 560 | 2005 | 37.3 |
| 5 | Patterns, correlates, and barriers to medication adherence among persons prescribed new treatments for HIV disease | Health Psychology | 485 | 2000 | 24.3 |
| 6 | HIV prevalence, risk behaviors, health care use, and mental health status of transgender persons: Implications for public health intervention | American Journal of Public Health | 460 | 2001 | 24.2 |
| 7 | Sociodemographic and psychological variables influencing adherence to antiretroviral therapy | AIDS | 449 | 1999 | 21.4 |
| 8 | Adherence to combination antiretroviral therapies in HIV patients of low health literacy | Journal of General Internal Medicine | 411 | 1999 | 19.6 |
| 9 | Alcohol use, drug use and alcohol-related problems among men who have sex with men: the Urban Men's Health Study | Addiction | 403 | 2001 | 21.2 |
| 10 | Explaining disparities in HIV infection among black and white men who have sex with men: a meta-analysis of HIV risk behaviors | AIDS | 380 | 2007 | 29.2 |
| 11 | Stressful neighborhoods and depression: A prospective study of the impact of neighborhood disorder | Journal of Health and Social Behavior | 372 | 2003 | 21.9 |
| 12 | Randomised, controlled, community-level HIV-prevention intervention for sexual-risk behaviour among homosexual men in US cities | Lancet | 345 | 1997 | 15.0 |
| 13 | Internalized stigma, discrimination, and depression among men and women living with HIV/AIDS in Cape Town, South Africa | Social Science & Medicine | 339 | 2007 | 26.1 |
| 14 | Impact of a patient-centered, computer-based health information/support system | American Journal of Preventive Medicine | 320 | 1999 | 15.2 |
| 15 | Impact of HIV-related stigma on treatment adherence: systematic review and meta-synthesis | Journal of The International AIDS Society | 315 | 2013 | 45.0 |
| 16 | Population-level HIV declines and behavioral risk avoidance in Uganda | Science | 315 | 2004 | 19.7 |
| 17 | HIV and Aging: State of Knowledge and Areas of Critical Need for Research. A Report to the NIH Office of AIDS Research by the HIV and Aging Working Group | JAIDS-Journal of Acquired Immune Deficiency Syndromes | 289 | 2012 | 36.1 |
| 18 | Progression to AIDS: The effects of stress, depressive symptoms, and social support | Psychosomatic Medicine | 269 | 1999 | 12.8 |
| 19 | Using respondent-driven sampling methodology for HIV biological and behavioral surveillance in international settings: A systematic review | AIDS & Behavior | 265 | 2008 | 22.1 |
| 20 | Comprehensive treatment of extensively drug-resistant tuberculosis | New England Journal of Medicine | 259 | 2008 | 21.6 |

**Supplemental Digital Content 4**


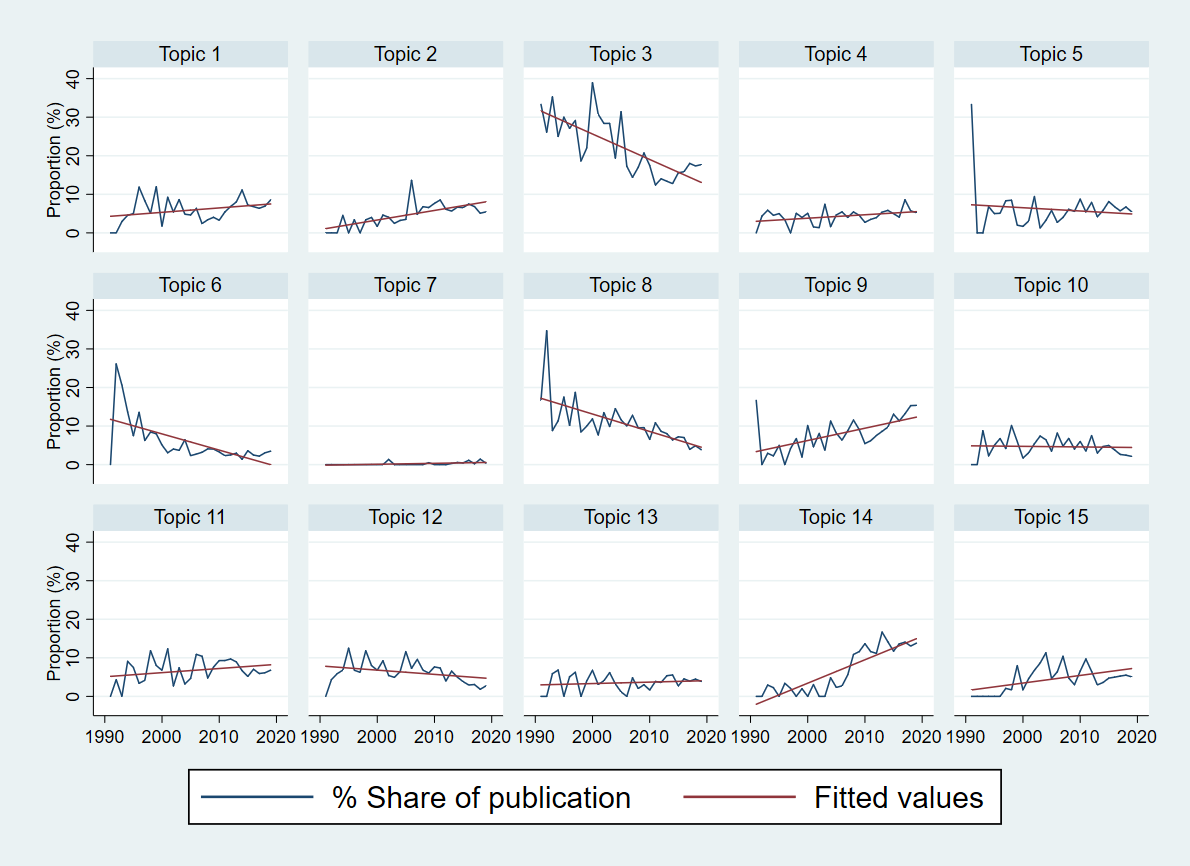

Supplement: Supplementary file 1 [file Table_1.DOCX]
